# Multidisciplinary Effort Leading to Effective Tuberculosis Community Outbreak Containment in Israel

**DOI:** 10.3390/microorganisms12081592

**Published:** 2024-08-05

**Authors:** Inbal Fuchs, Yelena Losev, Zohar Mor, Mor Rubinstein, Marina Polyakov, Tali Wagner, Tamar Gobay, Ester Bayene, Gila Mula, Hasia Kaidar-Shwartz, Zeev Dveyrin, Efrat Rorman, Ehud Kaliner, Sivan Haia Perl

**Affiliations:** 1 Clalit Health Services, Central District, Rishon LeTsiyon 7528809, Israel; inbalfu@clalit.org.il (I.F.); esteran@clalit.org.il (E.B.); gilamu@clalit.org.il (G.M.); 2 National Mycobacterium Reference Center, Ministry of Health, Tel Aviv 6810416, Israel; yelena.losev@phlta.health.gov.il (Y.L.); hasia.shwartz@phlta.health.gov.il (H.K.-S.); 3 Department of Tuberculosis and AIDS, Ministry of Health, Jerusalem 9438317, Israel; zohar.mor@moh.gov.il (Z.M.); tali.wagner@moh.gov.il (T.W.); 4 National Public Health Laboratories, Ministry of Health, Tel Aviv 6810416, Israel; mor.rub@phlta.health.gov.il (M.R.); zeev.dveyrin@phlta.health.gov.il (Z.D.); 5 Central District Department of Health, Ministry of Health, Ramla 7243003, Israel; marina.polyakov@rch.health.gov.il (M.P.); tamar.gobay@rch.health.gov.il (T.G.); ehud.kaliner@moh.gov.il (E.K.); 6 Ministry of Health Laboratories Department, Ministry of Health, Jerusalem 9134302, Israel; efrat.rorman@moh.gov.il

**Keywords:** tuberculosis, outbreak, Israel, whole genome sequencing, epidemiological investigation

## Abstract

Tuberculosis (TB) is the second-most prevalent cause of mortality resulting from infectious diseases worldwide. It is caused by bacteria belonging to the Mycobacterium tuberculosis complex (MTBC). In Israel, TB incidence is low, acknowledged by the WHO as being in a pre-elimination phase. Most cases occur among immigrants from high TB incidence regions like the Horn of Africa and the former Soviet Union (FSU), with occasional outbreaks. The outbreak described in this report occurred between 2018 and 2024, increasing the incidence rate of TB in the region. Control of this outbreak posed challenges due to factors including a diverse population (including Ethiopian immigrants, Israeli-born citizens, and immigrants from other countries), economic and social barriers, and hesitancy to disclose information. The unique multidisciplinary team formed to address these challenges, involving the local TB clinic, district health ministry, health maintenance organization (HMO) infectious disease consultant, neighborhood clinic, and National Mycobacterium Reference Laboratory (NMRL), achieved effective treatment and containment. Whole genome sequencing (WGS) proved pivotal in unraveling patient connections during the outbreak. It pinpointed those patients overlooked in initial field investigations, established connections between patients across different health departments, and uncovered the existence of two distinct clusters with separate transmission chains within the same neighborhood. This study underscores collaborative efforts across sectors that successfully contained a challenging outbreak.

## 1. Introduction

Tuberculosis ranks as the most fatal infectious disease globally. In 2022, the WHO reported 1.3 million TB-related deaths [1]. The disease is caused by the Mycobacterium tuberculosis complex (MTBC), a group of aerobic pathogens primarily affecting the lungs but capable of affecting virtually any organ [2].

TB disease is often curable with consistent and effective treatment. Standard treatment typically involves a regimen of several anti-tubercular antibiotics for a minimum of six months to target susceptible bacteria. Multidrug-resistant tuberculosis (MDR-TB) is characterized by bacteria resistant to rifampicin and isoniazid, the most effective first-line anti-TB drugs. Extensively drug-resistant tuberculosis (XDR-TB) refers to MDR-TB bacteria that is also resistant to any fluoroquinolone and either bedaquiline or linezolid. Treating MDR and XDR TB requires extended, expensive therapy and presents a substantial risk of relapse, treatment failure, prolonged transmission, and mortality, making them formidable challenges in TB elimination programs [1,3]. TB is transmitted through airborne infectious aerosols, making early detection crucial for preventing its spread. Effective intervention includes identifying TB disease cases early, potentially isolating affected patients, screening individuals exposed to the patient, and promptly administering preventive treatment. These measures are essential for minimizing community transmission of the disease [4].

In Israel, the incidence of TB has been on the decline over the past two decades. This decrease can be attributed to several factors, including changes in immigration patterns, pre-emigration screening of the Jewish Ethiopian population for TB, heightened awareness of TB risks, and the implementation of the National TB Program for control and elimination since 1997 [5,6,7,8]. To implement the program, the monitoring and treatment of TB in Israel involve multiple stakeholders who must collaborate synergistically, among them the Ministry of Health (MoH) Department for Tuberculosis and AIDS, health maintenance organizations (HMOs), Health Bureaus, hospitals, TB treatment centers, and the National Mycobacterium Reference Laboratory (NMRL). The Israeli MoH established nine specialized TB treatment centers throughout the country, allowing patients to choose a convenient location for their treatment. Israel has been recognized by the WHO as being in a pre-elimination phase, with a TB incidence rate of 2.3 cases per 100,000 population as of 2023.

Despite dedicated actions undertaken to control TB disease, occasional outbreaks still occur [7,9]. The majority of TB cases in Israel occur among immigrants from countries with high TB incidence rates, mainly the Horn of Africa (Ethiopia and Eritrea) and the former Soviet Union (FSU) [10].

Israel has observed a dramatic reduction in the TB incidence rates in the Ethiopian community compared to the first years following emigration (20/100,000 in 2020 compared to 60/100,000 in 1990) [data originated from the Israeli National Registry of TB, Department of Tuberculosis and AIDS, Ministry of Health, Israel]. The pre-emigration screening method selected by the Israeli MoH has been comprehensively described [7,11]. Briefly, Jewish individuals seeking to immigrate to Israel from Ethiopia undergo an initial tuberculin skin test (TST) and chest X-ray while still in Ethiopia. Active TB cases begin treatment before airlifting to Israel, whereas latent TB cases are treated after arrival [7]. 

During 2021, an uncommon cluster of cases was identified in a neighborhood in central Israel. Between August 2021 and August 2022, 14 new cases of TB were diagnosed in one primary healthcare center (Clalit HMO), serving 7049 residents of the same neighborhood, leading to an extensive epidemiological investigation. During the investigation, 43 cases were identified between June 2018 and January 2024, possibly related to the current outbreak, with an annual incidence of 138/100,000 (in comparison with the national incidence of 2.1/100,000). The synergy between field investigation and whole genome sequencing (WGS) epidemiological analysis proved pivotal in unraveling the links between all patients. It pinpointed an unlicensed bar in the neighborhood as a key infection hotspot, linked patients from other districts to the current outbreak, delineated two distinct, independent clusters among patients, and identified the index case. This descriptive study outlines an outbreak primarily affecting Israeli Ethiopians, illustrating collaborative efforts across various sectors, including local TB clinic staff, the team of the district MoH, the League of TB Prevention, the local municipality, and the NMRL team, aimed at containing such outbreaks.

## 2. Materials and Methods

### 2.1. Outbreak Report and Case Definition

A TB outbreak is defined according to the CDC as more TB cases than expected within a geographic area or population during a particular time period, and with evidence of recent transmission of M. tuberculosis among those cases [12]. 

TB cases were defined as patients diagnosed with either a positive culture or PCR from clinical specimens. Patients were also considered to have active TB and be related to the outbreak when a clinical diagnosis of TB was made (according to symptoms and radiological findings), but with no confirmatory laboratory test and with epidemiological links to another active outbreak case [13]. Symptoms of cough, hemoptysis, fever, night sweats, or weight loss were deemed to support the diagnosis of TB [14], with any pathological X-ray finding [15].

Bacterial isolates were defined as belonging to a cluster according to genotype. Strains with less than 12 differing single nucleotide polymorphisms (SNP) were considered to be in the same cluster [16].

### 2.2. Patient Data

Demographic and clinical data of the patients were obtained from the MoH Department for Tuberculosis and AIDS and gathered during the field epidemiological investigation [17]. Data included age at diagnosis, sex, country of birth, the year of immigration, risk factors (HIV coinfection and alcohol consumption), specimen type used for microbiological diagnosis, sputum smear positivity at diagnosis, site of infection (pulmonary versus non-pulmonary), and treatment outcome (treatment success, death, relapse). Treatment success is defined as completing a minimum of 80% of drug doses as prescribed, with evidence of bacteriological response and no evidence of failure [18,19].

### 2.3. Epidemiological Investigation Process

All patients were interviewed by an epidemiological nurse according to a structured questionnaire. All individuals who had contact with the patients beginning three months prior to the onset of symptoms were assessed (family, social, and work environments). Household contacts and close contacts were referred for further evaluation at a TB clinic and administered preventive treatment as necessary. The evaluation was performed using a two-step Mantoux test, with a cut-off of 5 mm or above being considered positive. All positive cases were then evaluated for symptoms (as mentioned above), physical examination, and chest X-ray. 

### 2.4. Laboratory Methods

#### 2.4.1. MTBC Identification and Drug Susceptibility Testing

All cultures processed in Israel were sent to the National Mycobacterium Reference Laboratory (NMRL), in Tel Aviv, Israel [7,8]. At the NMRL, smears from samples and TB cultures were stained using Ziehl-Neelsen staining and cultured on Lowenstein-Jensen (LJ) media and on a BACTEC MGIT 960 (BD, Sparks, MD, USA) system. All new, laboratory-confirmed TB strains were identified to the species level using a commercially available strip DNA probe test (Hain Lifesciences, Nehren, Germany) [20,21].

Drug susceptibility testing (DST) for first-line drugs was conducted at the NMRL using a BACTEC MGIT 960 assay. BACTEC MGIT 960 MDR strains and strains resistant to any three first-line drugs were further tested for second-line drugs using the resistance ratio method (RRM). 

#### 2.4.2. Whole Genome Sequencing and Bioinformatics

Whole genome sequencing (WGS) analysis enhances molecular susceptibility testing for both first-line and second-line drugs. Additionally, WGS is utilized for lineage determination and epidemiological investigations. The sequencing was conducted at the NMRL.

Genomic DNA from inactivated bacterial cultures was extracted according to the manufacturer’s protocol using a Maxwell RSC cultured cells DNA kit and the Maxwell^®^ 16 System (Promega, Madison, WI, USA) [22]. DNA paired-end libraries were prepared using an Illumina Nextera XT DNA Library Preparation Kit according to Illumina protocols. For sequencing, we utilized the Illumina MiSeq platform (Illumina, Inc.; Hayward, CA 94545 USA) using a MiSeq Reagent Kit v2 (500-cycles) (catalogue MS-102-2003) or a MiSeq Reagent Kit v3 (600-cycle) (catalogue MS-102-3003). Raw reads fastq files were quality analyzed by FastQC, and Kraken2 was used to identify mixed cultures [23]. MTBseq, a comprehensive pipeline for the WGS analysis of MTBC isolates, was used as the main tool for MTBC analysis of lineage, the identification of genomic variations, and cluster analysis [24]. Lineages were identified as part of the MTBseq pipeline, based on the SNP scheme developed by Coll et al. [25]. A cluster is defined as having an identical sequence or differing by fewer than 12 single nucleotide polymorphisms (SNPs) in the isolate sequence. A difference greater than 12 SNPs indicates a separate origin for the bacteria, thereby defining it as a distinct cluster. Genomic variations were compared to the WHO 2021 catalogue of drug-resistance-associated mutations in MTBC and used to identify those resistant to antibiotics [26].

#### 2.4.3. Data Availability Statement 

The data presented in this study are available on request from the corresponding author in consideration of patient data privacy.

### 2.5. Multidisciplinary Team for Outbreak Intervention

In order to plan and implement an intervention to contain the outbreak, a multidisciplinary team was established by the community-based infectious disease (ID) consultant of the Clalit HMO. The team included local TB clinic staff, the team of the district MoH (physicians and nurses), the local clinic in the neighborhood where the outbreak took place, including a nurse and a community worker of Ethiopian origin, the League of TB prevention, the local municipality, and the NMRL team. 

The goals of the multidisciplinary team were to improve communication between all parties involved, map the connections between known patients (by epidemiological as well as genetic tools), identify possible transmission sites in the community, and plan community intervention actions while bridging knowledge gaps, cultural diversity, and different belief systems.

### 2.6. Ethics Committee

An exemption from the need for ethics committee approval was obtained, since all data were routinely gathered in patient files.

## 3. Results 

### 3.1. Clinical and Epidemiological Characteristics

Between June 2018 and January 2024, 43 cases of TB were diagnosed in the outbreak community (Table 1, Supplementary Information Tables S1–S3). The annual incidence rate for the neighborhood in 2022, during which the outbreak peaked, was 138 cases per 100,000 population, in comparison with 2.1/100,000 for the rest of the country. A total of 33 patients received a microbiological confirmation of diagnosis (31 cases with positive culture, 2 cases with positive PCR), while 10 cases were diagnosed clinically (primarily children). A total of 33 patients were adults, with a mean age at diagnosis of 44 years. The majority of adult patients had pulmonary TB, and one patient had pleural TB. Among all pediatric cases, four had pulmonary infiltrates, and six had lymph node involvement. Two patients presented with failure to thrive.

Twenty-five cases (58%) were immigrants from Ethiopia, and all children were descendants of Ethiopian immigrants and were born in Israel (10, 23%). Five adult patients were Israeli-born (two of them to Ethiopian immigrants), and three additional adults were born elsewhere.

Out of 43 patients in the outbreak, 41 completed therapy and were cured according to the WHO definition [18], while 2 are still under treatment. Out of the cured cases, four had relapsed, and one died two years after treatment completion (the reason for death is not known). 

### 3.2. Field Epidemiological Contact Investigation Findings

During 2021, as the number of newly diagnosed cases in the neighborhood consistently elevated, there was increasing concern about further transmission. The initial case was a 54-year-old man who had migrated from Ethiopia 15 years earlier. There was no record of previous Mantoux testing or treatment for latent TB. In May 2021, he visited a hospital clinic with complaints of persistent cough, weight loss, fever, and night sweats. A chest X-ray showed cavitation, and a sputum sample tested positive on smear and culture for TB. He commenced a four-drug regimen and completed a nine-month course, eventually being cured. During contact tracing, he mentioned working in a non-licensed bar in his neighborhood but was hesitant to reveal its location or the identities of his clients.

**Figure 1:**
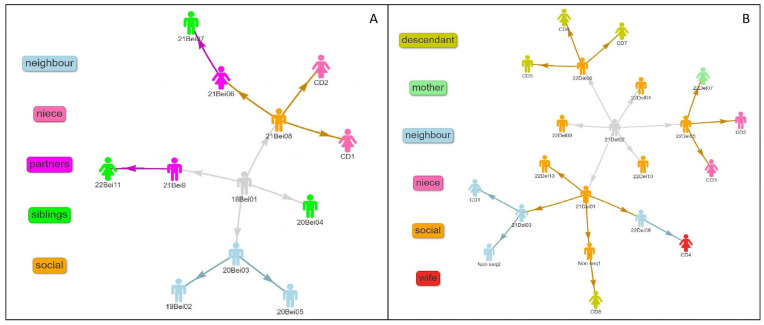
Contact tracing of diagnosed TB patients during TB outbreak: (**A**,**B**) show the network of diagnosed TB patients based on traditional field work contact tracing during TB outbreak in the neighborhood. Arrows indicate the most likely source case of infection, suggested by epidemiologic investigation findings. CD = clinical diagnosis; Non-seq = mycobacteria could not be genome sequenced. The figures in grey represent the index patients. Other colors indicate the characteristics of the contact with the source case, as detailed in the legend.

In parallel, several cases diagnosed during that year (21Bei06, 21Bei07, 21Bei08, 21Bei09, 21Bei10, 21Del01, 21Del02) (Figure 1) reported social alcohol drinking in such unlicensed bars in the neighborhood. During a field visit, one of these sites was found to be located in a small, unventilated space, owned by case 21Del02, who was consequently identified as the index case of this outbreak line. This data led to the identification of additional contacts who visited the place regularly (18Bei01, 21Del04, 21Del05, 21Del06), some of them whom were already symptomatic by that time. After establishing the makeshift bars as the main outbreak hypothesis, regular visits by the epidemiological nurse in the neighborhood were planned in order to identify other occasional visitors and possible contacts. 

During 2021, two cousin infants were diagnosed with TB. Attempts were focused on identifying the source case of the two babies, for whom the index case was not found. Childhood morbidity is a sentinel event in an outbreak since it indicates active transmission in the community, rather than reactivation of latent infection. Nevertheless, despite massive interrogations, the index case infecting both babies was not identified with regular field investigation. 

Additional two outbreak-related cases diagnosed during 2021 (21Del03, 22Del08) resided in the same building as the former cases, with no other social connections. This led to a vigorous investigation of all the neighbors in the residential buildings of the known cases and the identification of other contacts. 

The field investigation in combination with WGS identified 33 cases, which were categorized into two distinct transmission chains, as depicted in Figure 1 (Supplementary Information Tables S1 and S2). Another five cases were found to be related to the current outbreak by WGS only, and no epidemiologic links could be established by field investigation. Five cases, thought to be related at the beginning, were found to be unrelated to the current outbreak by the combination of the WGS and the filed investigation (Supplementary Information Table S3). 

All patients underwent an epidemiological investigation to identify all contacts (Supplementary Information Table S4). Close contacts underwent two-step Mantoux tests, and those who tested positive were referred to the clinic for anamnesis, a physical examination, and a chest X-ray. After ruling out active disease, rifampin or isoniazid prophylaxis was recommended for all contacts. For the 26 active cases across both clusters, contact investigation information is available for 18 of them. A total of 220 close contacts were identified, with 181 undergoing Mantoux testing. Of these, 105 (58%) tested positive. Additionally, 108 individuals had chest X-rays (three of whom had chest X-rays without a Mantoux test due to previously known positive results). Overall, 91 patients completed preventive therapy.

### 3.3. Whole Genome Sequencing to Identify Clustering and Genetic Lineages

**Figure 2:**
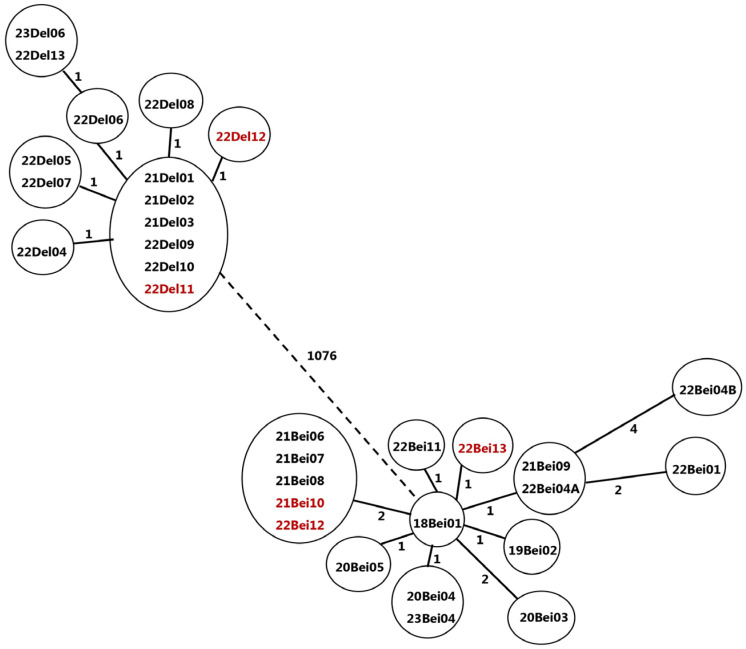
Minimum spanning tree of MTBC isolates relevant to the ongoing epidemiological investigation: The numerical values alongside the lines linking the circles represent the SNP numbers. A cluster of cases is defined when the distance between the isolates in the group is 12 SNPs or fewer. Identical isolates appear in a single circle. The tree shows two clusters, each originating from a different index case. Most of the patients shown in the tree live in the same neighborhood. The patients marked in red were linked to the clusters solely through WGS.

Following the WGS analysis and clustering of the patients, two different clusters were identified among the patients. One cluster belongs to the Delhi-CAS lineage, and the other one belongs to the Beijing lineage. Each cluster contains 13 patients (a few have more than 1 culture) (Figure 2). 

Five patients were only identified as part of the cluster using WGS and were not identified through field epidemiological work (marked in red in Figure 2). Moreover, genomic investigation revealed transmission lines that were not identified through traditional investigation tools. Among these was the case of the two infant cousins mentioned above. During interviews with the babies’ contacts, some of the neighbors mentioned a woman who had watched over both infants and had moved out of the building earlier that year. This woman was diagnosed with TB only after moving to another city and was therefore treated at a different TB clinic. Genotyping results indicated that she belonged to the neighborhood cluster, and deliberate, repeated questioning revealed the direct connection between the three of them. 

Two patients living in the neighborhood, initially thought to be part of the same cluster, were ultimately found not to be related following WGS analysis. Three additional cases could not undergo sequencing due to the absence of a positive culture. Field investigations did not establish any direct connection to other cases, thereby ruling out their classification as definite outbreak cases (Supplementary Table S3).

Phenotypic and genotypic susceptibility testing identified resistance to Streptomycin among patients from the Beijing cluster. A Lys43Arg mutation in the *rpsL* gene, known to confer Streptomycin resistance [27], was identified in all sequenced samples from this cluster. Cultures from Delhi-CAS patients were found to be susceptible to all the tested drugs. 

### 3.4. Multidisciplinary Team Interventions: TB Outbreak Response Plan

Joint interventions conducted by the taskforce included: the drafting of a local algorithm reflecting national and HMO services-based standards of care offering guidance in primary healthcare settings; academic activities in two hospitals with the infectious disease staff to raise awareness of TB symptoms and ensure the rapid identification and diagnosis of cases; a webinar targeted at primary clinics in the region (covering TB clinical awareness, diagnosis, and practical instructions for managing patients suspected of TB); an infectious disease-pulmonology conference; and a neighborhood awareness talk. The latter was given on a popular radio station in Amharic, conveying the contents of TB awareness based on the values and beliefs characteristic of the community. In May 2023, a community outreach event took place, organized by the same taskforce and municipal partners and financed by the league for TB prevention. The event included lectures providing knowledge about the disease and its prevention, a ceremonial speaker, health-promoting activities, and a question-and-answer panel. The event was accompanied by simultaneous translation into Amharic. It was advertised at the community centers in the neighborhood and at the local clinic, and it was attended by many of the neighborhood residents of all ages. 

Considering the makeshift bars hypothesis as the central locations of disease transmission, educational meetings with the local HMO clinic took place in order to help communicate messages to the community. The main principles were early referral to medical advice in cases of suspected symptoms, social distancing in cases of prolonged coughing, refraining from long stays in unventilated spaces, and encouraging a reduction in alcohol consumption. 

Intervention efforts, combined with the treatment of active cases, successfully contained the outbreak. In 2024, only one patient related to this outbreak was diagnosed, resulting in an incidence rate of 7 per 100,000, compared to 138 per 100,000 during the peak year of 2022.

## 4. Discussion

Between June 2018 and January 2024, a total of 43 TB cases were diagnosed in a single neighborhood, primarily among Ethiopian residents. Of these, 25 individuals were born in Ethiopia, while 15 were Israeli-born residents (12 of whom were descendants of Ethiopian immigrants), and an additional 3 were born elsewhere. This led to an extensive outbreak investigation. 

Ethiopian Jews have been immigrating to Israel for many years. According to data from the Central Bureau of Statistics in Israel, a total of 88,355 immigrants from Ethiopia arrived in Israel between 1984 and 2022 [6]. Commonly, upon arriving in Israel, members of the Ethiopian community remain for several months in an integration center, where they receive citizenship, preliminary Hebrew teaching, and centralized primary healthcare services, including latent tuberculosis infection (LTBI) preventive treatment when indicated. After leaving the centers, most new immigrants tend to reside in distinct neighborhoods close to other Ethiopian-born family and friends. 

Previous TB outbreaks in Israel, which occurred in integration centers, were recently analyzed and published [7]. Patient- and system-level contributing factors to outbreaks included nonadherence to latent TB treatment, staff miscommunication, poverty, alcohol and substance abuse, and challenges in coordinating transportation and medical coordination. Living in urban areas may also be a contributing factor to TB outbreaks, as mentioned earlier [28]. 

Ethiopian immigrants tend to live in close-knit communities where stigma about TB exists, affecting the willingness of patients to seek diagnosis and cooperate with investigations [29]. Additionally, the economic challenges faced by immigrants hinder their ability to seek TB diagnosis, as they may struggle to take time off work and face potential job loss [30]. These factors add to the complexity of outbreak treatment and underscore the importance of community involvement.

The currently studied outbreak occurred in a neighborhood in central Israel, outside of the integration center. Residents of this community are affiliated with various HMOs, with the local Ministry of Health responsible for information gathering and coordination. After initial diagnosis, TB cases are treated in designated TB clinics that are part of the national program of TB eradication, but the clinic is not physically located in the neighborhood and belongs to another HMO, which brings difficulties in terms of information sharing between different clinics and insurers. Other entities involved in the treatment of TB patients in the event of an outbreak are the Department of Tuberculosis and AIDS at the Ministry of Health, the local health bureau, and the epidemiological nurse team, and the NMRL. Thus, the formation of a multidisciplinary team was necessary, comprising representatives from each relevant sector. The team was guided by an ID consultant, who served as the main liaison between all parties. This approach ensures a thorough method for treatment and investigation, encompassing all relevant aspects. The multidisciplinary team acknowledged the necessity for intervention across two interconnected dimensions. Firstly, identify high-risk activities (such as alcohol consumption and social gatherings in small, poorly ventilated spaces) and populations at heightened risk for TB, and propose actionable measures to address them. Secondly, address gaps in information sharing among diverse stakeholders.

The team reached out to trusted community representatives who hold considerable influence among the residents. These individuals were knowledgeable about the cultural practices and beliefs of the community, resulting in enhanced collaboration among the patients. To foster cooperation among patients involved in this outbreak, it was essential to ensure the accessibility of information about the disease, treatment options, and risk factors to this population [31]. 

Continuous information transfer was required among the three main branches of outbreak management: the TB clinic, the MoH, and the laboratory. The multidisciplinary team ensured ongoing information sharing across all branches. Technical difficulties included the absence of a digital interface between the TB clinic and the primary care clinic, as well as a lack of digital accessibility to the findings of the epidemiological investigation for the primary care staff serving the outbreak community. Therefore, the infectious disease consultant diligently collected information from all pertinent sources to gain a comprehensive understanding of the outbreak’s elements.

Other issues addressed by the multidisciplinary team included identifying and providing solutions to economic and social barriers to TB treatment completion. Meetings with a social worker and a community health worker in the outbreak clinic were held, and financial support was provided as needed by the Tuberculosis Prevention League. In addition, a budget was provided to finance the patients’ transportation to the TB clinic when needed.

A lack of collaboration and unwillingness to disclose information about contacts were two of the main problems in the current outbreak. The information gathered from the field investigation, including daily searches in the neighborhood, helped identify an unventilated, unlicensed bar where social alcohol drinking took place, and where most of the transmission occurred. Crowded social gatherings in poorly ventilated, small spaces are well-known risk factors for triggering TB outbreaks [32,33,34].

Identification of the transmission lines during the field investigation was not complete, and several cases could not be related to source cases. The information obtained from WGS led to the completion of the epidemiological picture by adding several cases that were previously not known to be cluster related, as well as identifying the proximity of strains and revealing the transmission lines more accurately. Molecular epidemiology, and specifically WGS, has a well-established and critical role in delineating the extent of TB outbreaks [35] as well as evaluating the efficacy of TB-control efforts. Throughout the field investigation, the main outbreak hypothesis was that of transmission in bars and makeshift drinking places. At least 12 outbreak cases were known to drink alcohol in such places (known risk factor for TB, [30,36], hence assumed to belong to the same cluster). WGS revealed this assumption to be wrong, as those 12 cases were divided into two different genetic clusters of distinct lineages. The outbreak was discovered to be actually a combination of two different outbreaks that merged, with two index cases and transmission lines. The results of the genetic investigation also provided information on additional patients in different parts of Israel who belonged to these two clusters, helping to better characterize the chain of transmission and other contacts. 

Data generated from molecular analysis must be communicated thoughtfully to guide public health measures aimed at reducing disease transmission. Effective laboratory surveillance requires close communication among laboratories, public health workers, and institutional infectious disease experts. These experts must then engage and empower the community to participate in control activities.

TB strains can be divided into various lineages according to their DNA patterns. The geographical prevalence of each lineage is usually common, although it is not true for some of them nowadays due to the massive migration between countries [37]. The two most common lineages in Israel in the past few years are Beijing and Delhi-CAS [10,38]. The Beijing lineage is globally distributed. It has a relatively high transmission rate, an increased drug resistance rate, and is associated with outbreaks [37,39]. It was suggested that the Beijing strain has a greater risk for unsuccessful treatment and relapse among several populations [40]. Our previous study, which focused on TB in Israel in 2021 [10], showed that the Beijing lineage in Israel is indeed represented by strains resistant to all first-line anti-TB drugs, separately and in combination. Here, the Beijing cluster is resistant to Streptomycin, which is the most prevalent drug to which the TB strains in the previous study were resistant (18 out of 26 strains). Regarding the country of origin of the patients, in 2021, patients with Beijing lineages originated from many different geographical areas, such as FSU, Africa, Europe, Asia, etc. In this study, the index case of the cluster of cases with the Beijing lineage strain was from Ethiopia. 

A large study from 2019 showed that the CAS lineage is mainly distributed in South Asia, the Middle East, and East Africa [41]. There is clear evidence of its presence in Ethiopia [42]. In this outbreak, the index case is from Ethiopia. According to our previous study, Delhi-CAS exhibited a very low resistance rate, in contrast to other studies on this lineage [10,41]. The current cluster is susceptible to any drugs tested.

## 5. Conclusions

The current outbreak is likely the result of several factors, including being a member of a close ethnic community, alcohol abuse, TB stigma, social gatherings in small, poorly ventilated spaces, and low economic status. Addressing these factors is crucial to fostering willingness within the Ethiopian community to seek diagnosis and treatment for TB, thereby reducing the risk of further outbreaks. The utilization of WGS data provided by the reference laboratory, combined with collaboration among all stakeholders coordinated by a multidisciplinary team, facilitated the identification of previously unrecognized connections between patients from different health departments and contributed to containing the outbreak. The effective control of a large TB outbreak requires constant collaboration efforts between different stakeholders.

## Figures and Tables

**Table 1:** Epidemiological and clinical data of 43 cases of a TB outbreak in central Israel.

Characteristic	Cluster A (13)	Cluster B (13)	No Genetic Sequencing (17)
Age (mean) ± SD	38 (±15.6)	48 (±19.3)	22 (±23.8)
Children n (%)	0	0	10 (59%)
Female sex n (%)	5 (38%)	2 (15%)	13 (76%)
Country of birth			
Ethiopia	8 (62%)	11 (85%)	6 (35%)
Israel	3 (23%)	1 (7.5%)	11 (65%)
Other	2 (15%)	1 (7.5%)	0 (0%)
TB site			
Pulmonary	13 (100%)	13 (100%)	10 (59%)
Pleural	0 (0%)	0 (0%)	1 (6%)
Lymph nodes	0 (0%)	0 (0%)	6 (35%)
Sputum smear positive at diagnosis	8 (61%)	7 (54%)	1 (6%)
Culture positive	13 (100%)	13 (100%)	5 (30%)
Treatment outcome			
Death	1 (8%)	0 (0%)	0 (0%)
Success of treatment	13 (100%)	11 (85%)	17 (100%)
relapse	3 (23%)	1 (7.5%)	0 (0%)
Risk factors			
HIV infection	0 (0%)	2 (15%)	1 (6%)
Alcohol consumption	4 (31%)	8 (62%)	1 (7%)

## Data Availability

The data presented in this study are available on request from the corresponding author in consideration of patient data privacy.

## Supplementary Materials

The following supporting information can be downloaded at: https://www.mdpi.com/article/10.3390/microorganisms12081592/s1. Table S1: Demographic information of patients and drug susceptibility of their microbial TB samples. The first two digits in each microbial sample name designate the sampling year (i.e. 18 indicates 2018). The phenotypic susceptibility column indicates drugs to which the test results where resistant (R). Demographic information of patients and drug susceptibility of their microbial TB samples; Table S2: The first two digits in each microbial sample name designate the sampling year (i.e. 18 indicates 2018). The phenotypic susceptibility column indicates drugs to which the test results where resistant (R); Table S3: Demographic information of patients without genomic sequencing. Non-seq = bacterial isolate could not be sequenced due to technical issues. CD = clinical diagnosis, letter in brackets indicates cluster pertinence. NR = not related to any cluster by field and genomic investigation; Table S4: Contact investigation of active TB cases results. The data includes contact investigation results for 18 out of 26 patients in clusters A and B, with information unavailable for the remaining 8 patients. Among the 17 patients without genomic sequencing, 10 were children (thus no contact investigation was conducted), 5 were determined to be unrelated to the outbreak, one patient was culture-negative, and another did not cooperate with the investigation.
